# Mitochondrial Haplotypes Influence Metabolic Traits in Porcine Transmitochondrial Cybrids

**DOI:** 10.1038/srep13118

**Published:** 2015-08-19

**Authors:** Guanghui Yu, Hai Xiang, Jianhui Tian, Jingdong Yin, Carl A. Pinkert, Qiuyan Li, Xingbo Zhao

**Affiliations:** 1 National Engineering Laboratory for Animal Breeding; Ministry of Agricultural Key Laboratory of Animal Genetics, Breeding and Reproduction; College of Animal Science and Technology, China Agricultural University, 100193, Beijing, China; 2 State Key Laboratory of Animal Nutrition, College of Animal Science and Technology, China Agricultural University, 100193, Beijing, China; 3 Department of Pathobiology, College of Veterinary Medicine, Auburn University, Auburn, AL 36849, and Department of Biological Sciences, College of Arts and Sciences, The University of Alabama, Tuscaloosa, AL 35487, USA; 4 State Key Laboratory for Agribiotechnology; College of Biological Sciences, China Agricultural University, 100193, Beijing, China

## Abstract

In farm animals, mitochondrial DNA mutations exist widely across breeds and individuals. In order to identify differences among mtDNA haplotypes, two porcine transmitochondrial cybrids were generated by fusion of a Lantang pig cell line devoid of mitochondrial DNA with enucleated cytoplasm from either a Large White pig or a Xiang pig harboring potentially divergent mitochondrial haplotypes. These cybrid cells were subjected to mitochondrial genome sequencing, copy number detecting and analysis of biochemical traits including succinate dehydrogenase (SDH) activity, ATP content and susceptibility to reactive oxygen species (ROS). The Lantang and Xiang mitochondrial genomes were highly homologous with only 18 polymorphic sites, and differed radically from the Large White with 201 and 198 mutations respectively. The Large White and Xiang cybrids exhibited similar mtDNA copy numbers and different values among biochemical traits, generated greater ROS production (*P* < *0.05*) and less SDH activity (*P* < *0.05*) and a lesser ATP content (*P* < *0.05*). The results show that functional differences exist between cybrid cells which differ in mitochondrial genomic background. In conclusion, transmitochondrial cybrids provide the first direct evidence on pig biochemical traits linking different mitochondrial genome haplotypes.

The mitochondrial genome plays an essential role in energy production, and involves the control of many cell functions including oxidative phosphorylation (OXPHOS), reactive oxygen species (ROS) production, thermogenesis, cellular calcium homeostasis and regulation of apoptosis. The mitochondrial DNA (mtDNA) variation was reported in association with a wide spectrum of human traits, either as specific mutations or deletions leading to metabolic and developmental disorders, or as multiple factors in complex traits, including diseases, athletic performance, body fat mass, metabolic traits and lifespan of humans and other vertebrates^1^,^2^,^3^.

In farm animals, mtDNA variations were reported in association with complex traits in pigs^4^,^5^, cattle^6^,^7^,^8^, chickens^9^, ducks^10^ and donkeys^11^. However, the phenotypic effects of variations in the mitochondrial genome were difficult to isolate owing to confounding variation in the nuclear genome, epigenetic phenomena, and environmental factors. Few animal models have been available for directly investigating the effect of mtDNA variations on complex metabolic phenotypes *in vivo* until the creation of transmitochondrial cybrids^12^. The transmitochondrial cybrid is the fusion of a cytoplast (enucleated cell) to an intact cell (often ρ^0^) to produce a single cell with mixed mitochondrial populations^13^,^14^. The cybrid allows characterization of the biochemical phenotypes of cell lines, and this approach also provides a uniform nuclear background for studies using cytoplasts made from different individuals, eliminating confounding nuclear variables^15^.

In this study, we isolated somatic cells from three pig breeds representing different mitochondrial genome haplotypes: Lantang (C0), Xiang (C1) and Large White (C2). Their mitochondrial DNAs and variations were then characterized. Transmitochondrial cybrids were generated by mitochondria: nuclear transfer using C0 nuclei and mitochondria of either C1 or C2 plasma (schematic diagram see Fig. 1). Using cybrids, mitochondrial respiratory capacity (ATP content and SDH activity) and oxidative stress (ROS production) were compared as measures of energy metabolism. The aim of this study was to shed light on the influences of mitochondrial haplotype by creating an *in vivo* cellular model. The model would allow separation of phenotypic changes resulting from mitochondrial genome variation from confounding influences imparted by nuclear gene expression, epigenetic phenomena and environmental factors. This study provides the first evidence detailing the mtDNA effect on targeted biochemical traits in pig cells.

## Results

### Features of porcine cybrids

In order to introduce selective markers for recipient cells devoid of mitochondrial DNA (ρ0 cells), a *GFP*-*neo* gene was introduced into C1 cell line, with a G418 selectable marker recognized under fluorescence (Fig. 2). Mitochondria donor cells (C0, C1 and C2) were transfected by MT-RFP which stained mitochondria temporally (10 days) with a red fluorescence marker (Fig. 2). C0 cells harboring GFP-*neo* gene were used to create ρ0 cells by treatment with R6-G. The ρ0 cell formation was thin and flat (Fig. 2A,b1), and needed uridine and pyruvate as essential nutrients. We evaluated the ρ0 effect of mitochondria dysfunction by removing uridine/pyruvate from the culture medium. With 5% dialyzed FBS supplementation, ρ0 cells gradually died after 1 week post-treatment. Confirmation was obtained by Janus green B (JG-B) staining which labels specifically vital mitochondria by reduction and oxidation of the electron transfer chain alteration^16^. Our results indicated that ρ0 cells were free of staining with JG-B (Fig. 2A,b3) while the normal control cells were viable based on staining profile (Fig. 2A,b2). Mitochondria donor cells transfected with MT-RFP were enucleated by treatment of cytochalasin B and centrifugation. Enucleation was confirmed by Giemsa staining (Fig. 2A,c1–c3). Pig cybrids were created by PEG fusion; unfused cells died under G418 selection in the absence of uridine and pyruvate. As shown in Fig. 2A(d1–d3), only fused cells (cybrids) containing exogenous mitochondria and a transfected *GFP*-*neo* nucleus survived in the selective medium. Cybrid mitochondria were labeled with MT-RFP and nuclei with GFP, and these reporter markers enabled detection under fluorescence (Fig. 2A,e1–e3).

In order to verify cybrids containing the proper introduced/exogenous mtDNA, we identified cybrid mtDNA D-loop regions using PCR direct sequencing. Results indicated that cybrids contained the mtDNA derived from Xiang or Large White breeds, as well as detectable Lantang mtDNA. As with the control, 2 weeks after cell fusion, cybrids presented heteroplasmic polymorphisms which contained both endogenous and exogenous mtDNA. These heteroplasmic mtDNAs persisted in mitochondria, but gradually degraded to where the endogenous host mtDNAs were no longer detectable in culture after 4 weeks (Fig. 2B).

### Identification of the mitogenome sequences of primary cells and cybrids

The complete mitogenome sequences of the three pig cells (C0, C1 and C2) and the cybrids (C0-C0, C0-C1 and C0-C2) were acquired by amplification and PCR direct sequencing and deposited in GenBank with accession numbers of KC250273–KC250275 for C1, C0 and C2. Not surprisingly, C0-C0, C0-C1 and C0-C2 sequences were consistent with background sequences and those noted in the literature. C0 and C1 presented higher homology, through the complete mitochondrial genome. There were 18 mutations which included only 6 missense mutations and 3 synonymous mutations for coding peptides, and 1 mutation in 16S rRNA, 2 mutations in tRNAs and 6 mutations in D-loop sequences. All six missense mutations between C0 and C1 exhibited lower Grantham scores (<100) which indicated conservative (score 0 to 50) or moderately conservative (score 51 to 100) substitutions according to the classification proposed by Li *et al.*^17^. However, C0/C2 and C1/C2 comparisons reflected much greater diversity; 201 and 198 mutations respectively, spread across the entire mitochondrial genome. C2 held one moderately radical (score 101 to 150) and one radical (>150) substitution when comparing to C0 and C1. Ser246Phe in ND1 and Leu63Ser in ATP8 were noted, hinting to a greater difference across C2 and C0 or C1 mitogenomes. Missense mutations of mitogenomes from C0, C1 and C2 were listed in Table 1 and detailed mutation data can be found in Table S2 (red font).

The extant mitochondrial control region sequences (2 sequences for Xiang, 1 for Lantang and 17 for Large White, respectively) and the mitogenome sequences of Xiang (1 sequence) and Large White (5 sequences) in GenBank database were used. A consensus NJ tree was constructed along with the three pig sequences determined in this study by complete control region sequences (Fig. 3A) and one tree was constructed by mitogenome sequences (Fig. 3B). Both trees revealed a divergence among pigs, which clustered into two groups; either mixed sequences of the three breeds (Large White, Lantang and Xiang pigs) or pure Large Whites (Fig. 3A,B). The results showed that the C2 mitochondrial DNA genome sequence derived from the Large White mitochondria donor, was far from the mitogenome sequences of the other two mitochondria donor cells (C0 and C1). These findings suggest that Large White pigs were largely crossed as paternal parents with Chinese native pigs; however, C2 maintained its pure Large White maternal genetic characteristics, which exhibited developmental differences consistent with Chinese native pigs.

### Identification of mitochondrial DNA copy numbers in primary cells and cybrids

Mitochondrial DNA abundance was measured in primary cells and cybrids, Not surprisingly, in skeletal muscle satellite cell line, C0 had higher mtDNA copies than other cells (*P* < *0.01*), which harbored similar mtDNA copy number (*P* > *0.05*) (Fig. 4). Comparable mtDNA copy numbers were identified by comparison to a one-copy and nuclear-encoded GADPH gene.

### Comparison of SDH activity, ATP content and ROS production in different cells

Both SDH activity and ATP content were used as positive indicators of mitochondrial respiratory capacity. SDH activity was regarded as a marker enzyme for mitochondrial function, especially for OXPHOS enzymes and electron transport chain (ETC) in cellular energy metabolism. ATP is a high-energy phosphate compounds used as a source of chemical energy, it storages and discharges energy through the mutual conversion with ADP, thus ensure energy supplementation for cellular function. Generation of ROS, produced during cell metabolism, leads to oxidative stress and subsequent apoptosis.

In this study, measurement of SDH activity, ATP content and ROS production proved similar in both primary cells and cybrid cells (Fig. 5). Amongst primary cells, C0 and C1 cells exhibited similar concentrations of SDH which were higher (*P* < *0.05*) than those observed in C2 cells. For ATP content, C0 was highest (*P* < *0.05*), with C1 at an intermediate concentration followed by C2 (*P* < *0.05*). Compared with primary cells and cybrid cells, ρ0 cell (C0/R-6G, C1/R-6G and C2/R-6GL) declines on SDH level and ATP content were putatively associated with mitochondrial OXPHOS deficits/dysfunction (*P* < *0.05*). ROS production in C0 and C1 was similar, but both were lower in concentration than that in C2 (*P* < *0.05*), and the ρ0 cells (C0/R-6G, C1/R-6G and C2/R-6GL) exhibited higher values (P < 0.05).

By comparing cybrids with C0-C0 control, the results of SDH activity, ATP content and ROS production measurements illustrated that cybrids with exogenous mitochondria could readily recover mitochondrial function and normal cellular metabolism. Whether observing SDH activity, ATP content or ROS production, C0 ρ0 cells fused with C1’s mitochondria always recovered to a greater degree than that fusion with C2’s mitochondria (Fig. 5). For either SDH activity or ATP content, combinations of C0 + C1 were higher than the corresponding combinations of C0 + C2 (*P* < *0.05*). Lastly, ROS content of C0 + C1 was lower than that of C0 + C2 (*P* < *0.05*).

## Discussion

The pig is one of the most important farm animals for meat production worldwide. After the first divergence between European and Asia wild boars, pigs were domesticated independently across Eurasia with rare transcontinental genetic interchanges in the early ages. Subsequent divergence occured across two subgroups (European and Asian types)^18^,^19^. In recent centuries, pigs were raised under various artificial selection practices in different geographical regions. Thus, cultivated pig breeds possess specific characteristics, for example, European commercial breeds (such as Large White, Landrace, Duroc) have stronger quantitative trait loci imparting faster growth and feed efficiency/conversion rates but with less-desirable meat quality, lower reproductive performance and less stress tolerance in comparison to Chinese indigenous breeds (such as Xiang, Jinhua, Taihu, and Lantang breeds). However, under conventional selection practices targeting improved production performance, cross-breeding efforts between European commercial breeds and Chinese native breeds has become more commonplace^20^,^21^. Typically, European males (*e.g.*, Large White breed) are used in such cross-breeding efforts with Meishan females or other prolific Chinese native breeds, to generate F1 hybrid offspring. Therefore, it was not surprising to find Large White genetics within mitochondrial populations within Chinese indigenous pig breed as was noted with other European breed sequences^21^,^22^.

In this study, differences in biochemical traits (SDH activity, ATP content and ROS production) were detected in different porcine mtDNA haplotypes. C2 cells generated more ROS along with lower SDH activity and ATP content in comparison to both C0 and C1 cells (Fig. 5). Both SDH activities and ATP content reflected mitochondrial metabolic capacity, while ROS production, indicated the level of oxidative stress *in vivo*.

For mitogenome sequences, C0 and C1 had similar sequences which differed for only 18 nucleotide substitutions and no radical substitutions for the missense mutations. While C0/C2 and C1/C2 comparisons presented large differences with 201 and 198 mutations respectively, including 2 radical missense mutations (Ser246Phe in ND1 and Leu63Ser in ATP8).

For mtDNA concentrations, all cybrids presented similar mitochondrial genome copy numbers. The results demonstrate that differences in biochemical traits (SDH activity, ATP content and ROS production) are contributed to the different mtDNA haplotypes, which is radically different between C1 and C2.

The nuclear genome and mito-nuclear interactions are known to be metabolically and physiologically important, and we speculated that mitochondrial genome variations were partially responsible for the differences in the measured biochemical traits of these three prototypical cell lines.

The potential role of mtDNA impacting domestic animal economic traits was postulated for several decades and was illustrated from a molecular perspective with the first estimation of cytoplasmic effects on production traits in dairy cattle^23^. The question remained open with some ambiguity in accumulated studies of mtDNA variations and associated reports in animal production^8^,^24^. Because of the importance of mitochondrial capacity and relevance of mtDNA polymorphisms and mutations identified in humans, animal scientists characterized a number of species-specific mitochondrial haplotypes and attempted to identify relevant production endpoints in an effort to better understand the underlying biology^10^,^15^,^24^,^25^,^26^. In the present study, we successfully created transmitochondrial cybrids based on porcine-specific mtDNA haplotypes using common nuclear genetics of the Lantang pig breed. Pig cybrids exhibited different biochemical traits including SDH activity, ATP concentration and ROS production. C2 cybrid (C0 + C2), which harbored large spans of divergent mtDNA sequence from that of both Lantang and Xiang, also exhibited greater ROS production and both lower SDH activity and ATP content in comparison to the cybrids of C1 (C0 + C1). The creation of cybrids isolated phenotypic variation in the mitochondrial genome from confounding influences of nuclear genetics, epigenetic phenomena, and environmental factors. Accordingly, this model provided a new cell culture-based system for directly investigating the effects of mtDNA variants on complex metabolic phenotypes *in vivo*^27^.

The cybrids provide the first *in vitro* mitochondrial modeling system to evaluate biochemical indicators associated with biochemical traits of relevance to specific mtDNA haplotypes. This technology provides an initial step in characterizing potentially mtDNA effect associated with complex traits. Ultimately, the ability to harness metabolic traits of economic importance will depend on the generation of transmitochondrial swine (mitopig model). Such genetic engineering efforts will further our understanding of the mitochondrial genome’s impact on economic traits and provide future mtDNA genotype selection end points in animal breeding programs.

## Methods

### Animals and cell lines

The pigs used in this study were maintained under the standard management conditions. The guidelines of the experimental animal management of China Agricultural University (CAU) were followed throughout the study, and the experimental protocols were approved by the Experimental Animal Care and Use Committee of CAU. Pigs were housed under routine veterinary care and were humanely sacrificed to prepare samples for DNA isolation and cell differentiation.

Somatic cells were isolated from three pigs: C1, C2 and C0 from Xiang, Large White and Lantang breeds, respectively. C1 and C2 were derived from primary fetal fibroblasts and C0 from skeletal muscle satellite cells. Using Lipofectamine 2000 (Invitrogen), C0 was introduced a *GFP-neo* gene by a neomycin resistant expression vector pAP1^28^, which enables unambiguous staining in fluorescence. C1, C2 and C0 were transfected by CellLight^®^ Mitochondria-RFP (Invitrogen) which tags mitochondria with a red fluorescence marker.

### Creation of porcine transmitochondrial cybrids

**Figure 1:**
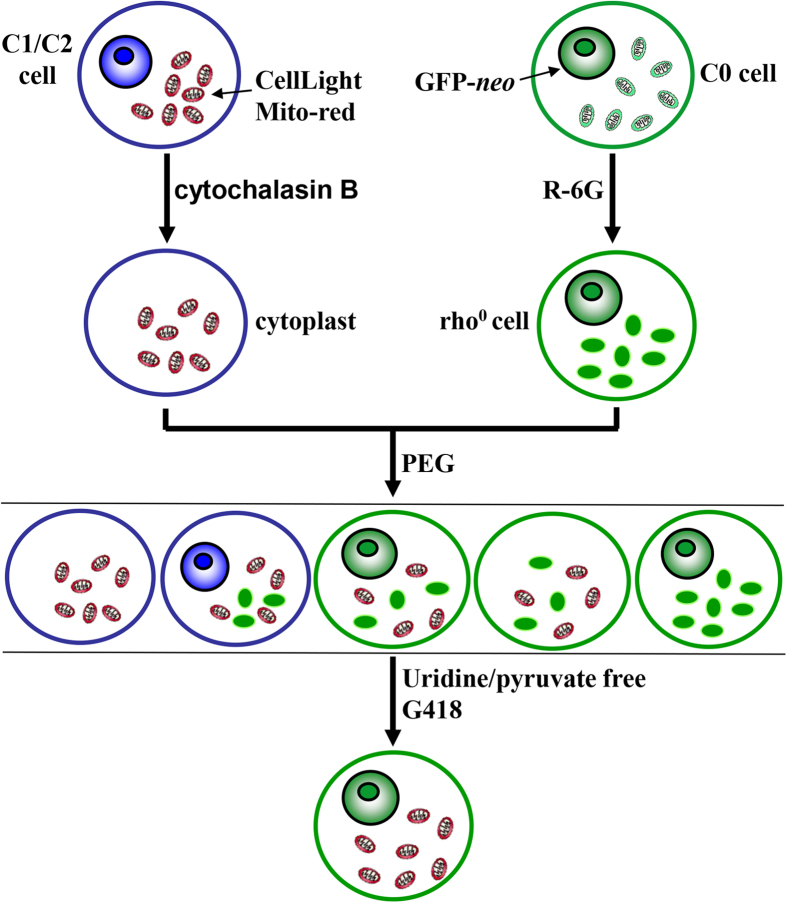
Schematic representation of the porcine cybrid construction. Mitochondria from C0, C1 and C2 were labeled in red by MT-RFP. C0 nuclei were transfected with GFP-*neo* gene that provided fluorescence recognition and G418 resistance for negative selection. After fusion, only cybrids survive in selection medium.

**Figure 2:**
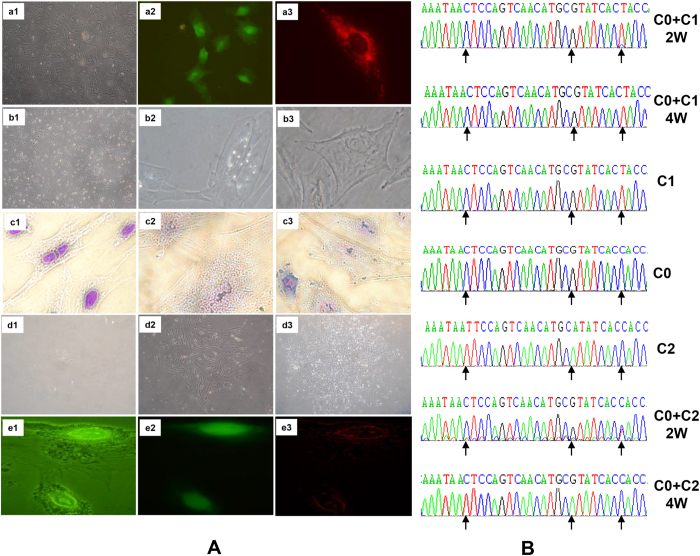
Porcine cybrid cell formation and verification. In (**A**) Cell transfection (a1–a3). a1, C0 cells (×40); a2, GFP-neo transfected C0 cells under fluorescence (×200, excitation/emission (nm): 555/584); a3, MT-RFP transfected C1 and C2 cells under fluorescence (×400, excitation/emission (nm): 488/510). b1–b3 indicated the JG-B detection of intact cells and ρ0 cells. b1, ρ0 cells cultured without uridine and pyruvate supplemented with dialyzed FBS (×40); b2, C0 cells transfected with GFP-neo stained using Janus Green B (×400); b3, ρ0 cells cultured with uridine and pyruvate supplemented (×400). c1–c3, enucleation of mitochondria donor cells (C1 and C2). c1, normal cells stained by Giemsa (×200). c2 and c3, enucleated cells after Giemsa staining (×400), here an intact cell (with nucleus) in c3 served as a control. d1–d3, Formation of pig cybrids. d1, cybrids in the first week where only a few cybrids survived in selection medium; d2, Week 2, clones gradually formed in selection medium; d3, cybrids at week 4; a large number of clones formed. e1–e3, cybrid detection under fluorescence; e1, cybrids under light microscopy (×400); e2, GFP expression under green fluorescence in transfected cybrids (×400, excitation/emission (nm): 488/510); e3, a transfected RFP gene was expressed in mitochondria and detectable under red fluorescence (×400, excitation/emission (nm): 555⁄584). (**B**) Sequence verification of mitochondrial origins in different cybrid cells. C0, C1 and C2 exhibit characteristic sequence signatures within the D-loop region, and cybrids (C0 + C1/2 W and C0 + C2/2 W) at the second week were characterized (double peaks with endogenous and exogenous mtDNA, C0 + C2/2 W with baseline discrimination). Heteroplasmic mtDNAs gradually degraded through the fourth week of cell culture after loss of baseline (sequencing noise) to where presence was no longer detectable. In cybrid cells, the acronym C0 denotes a common nuclear of Lantang primary cell, and +C0, +C1 or +C2 represent the source of mitochondria. 2 W, 4 W: cell culture for 2 weeks and 4 weeks, respectively. Arrows indicated specific mutations for a particular mtDNA haplotype.

**Figure 3:**
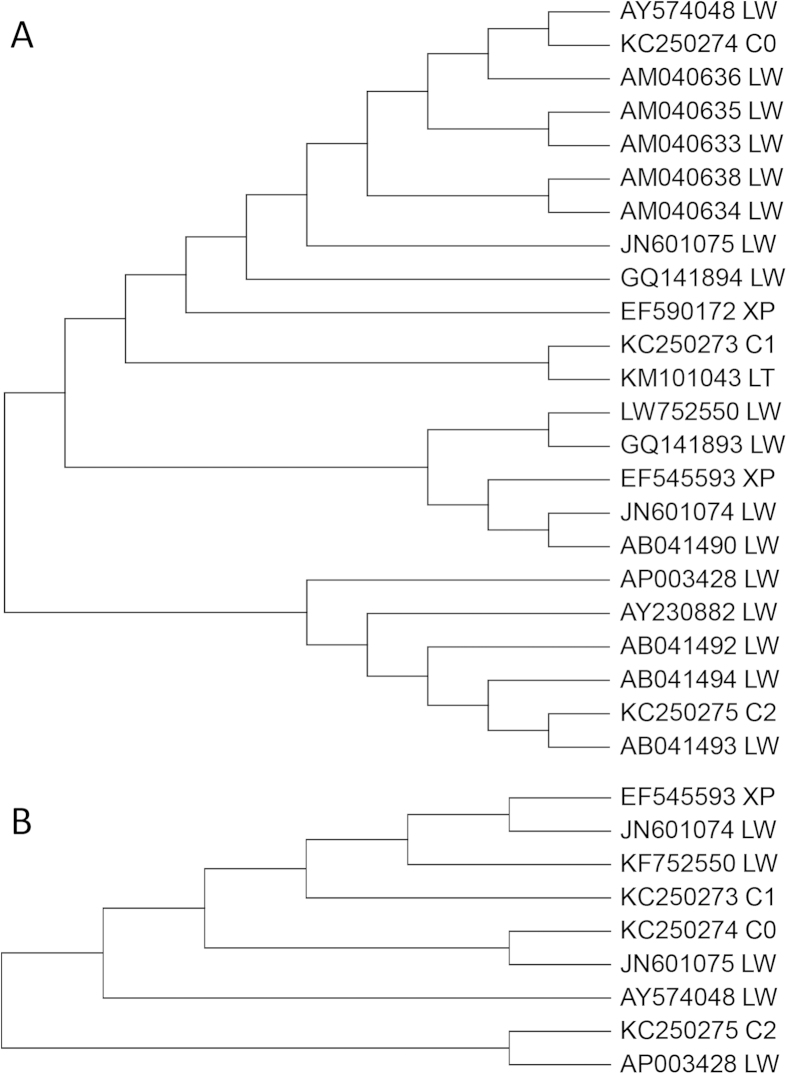
Relationship of Lantang, Xiang and Large White pigs revealed by mitochondrial control region and mitogenome sequences. Codes indicating GenBank entrires, 2-letter codes indicating breed origin.

**Figure 4:**
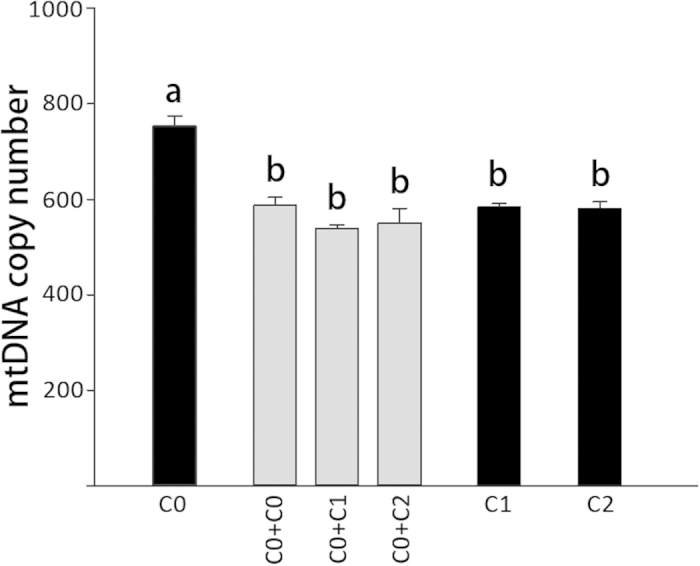
Measurement of mtDNA quantities in primary cells and cybrids. The black columns indicate primary cells; and the grey columns indicate cybrid cells. The letters above columns show the quantitative disparity (*P* < 0.05) between different cells.

**Figure 5:**
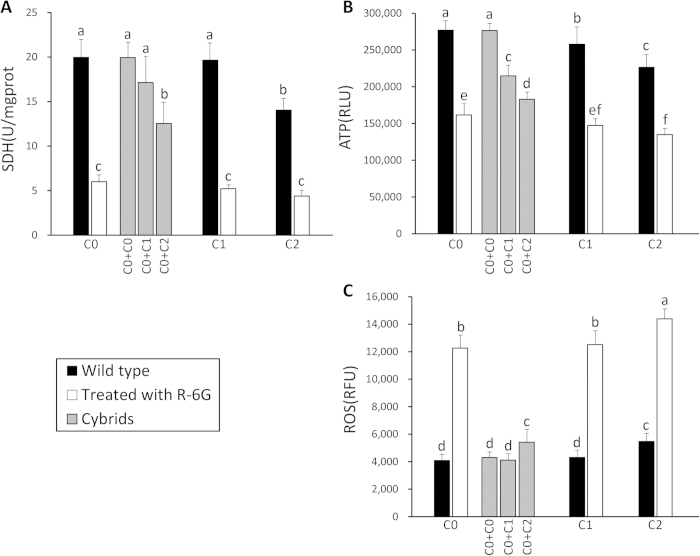
Comparison of SDH activity, ATP content and ROS production. The black columns indicate the isolated and cultured control cells without any special treatments; the white columns indicate mitochondria inactivated cells (ρ0 cells; treated with R-6G), and the gray columns indicate cybrid cells. The letters above columns show the quantitative disparity (*P* < 0.05) between different cells.

The *GFP-neo*-labelled C0 was used as nucleus donor (ρ0 cell) which was treated with rhodamine 6G (R-6G) (Sigma) at 5 μg/ml for 8 days^29^. C1, C2 and C0 cells were enucleated as mitochondria donors (enucleated cytoplasts) by centrifugation in the presence of 10 μg/ml cytochalasin B (CB) (Sigma). A total of 10^6^ ρ0 cells and 10^6^ enucleated cytoplasts were fused with 45% polyethylene glycol (PEG) (MW 1450; Sigma), and selected by G418 medium with free of uridine and sodium pyruvate. Mitochondria were stained by 1/5000 w/v Janus Green B (Sigma) and nuclei by Giemsa working solution (AppliChem), and all cells were observed under microscopy at ×400 magnification. The procedure is outlined in Fig. 1.

### Measurement of biochemical traits

SDH activity was measured by a succinate dehydrogenase assay kit (Jiancheng Bioengineer Institute, China). Briefly, cells in a 60-mm culture plate were trypsinized and harvested in 250 μL DPBS. Cell suspensions (100 μL) were added to a cuvette, and then added the reaction solutions according to manufacturer instructions. The OD value was recorded using spectrophotometer at 600 nm. Another 100 μL cell suspension was used for protein quantification by BCA Protein Assay Kit (CWBiotech, China). The SDH activity (U/mgprot) = (OD1-OD2)/[0.01* protein (mg) in the 100 μL cell suspension]. Each cell line was repeated three times.

Cellular ATP content was measured using an ATP Assay Kit (Beyotime, China). Total protein was assayed using BCA Protein Assay Kit (CWBiotech, China). Cells within a 35-mm culture plate were lysed; the lysate was then centrifuged and harvested. The relative light unit (RLU) value was detected using a luminometer module (Tecan F200; Tecan, Austria). In order to eliminate error in cell numbers and protein concentration, the lysate was divided into two equal parts; one was used to measure ATP content and the other for protein quantification using a BCA Protein Assay Kit (CWBiotech, China). ATP content was adjusted by protein concentration determined via the Bradford method^30^. Each cell line was repeated three times.

ROS content was measured using 6-chloromethyl-2′, 7′-dichlorodihydrofluorescein diacetate, acetyl ester (CM-H_2_DCFDA) (Genmed Scientific, US). Cells in a 60-mm culture plate were trypsinized and 10^6^ cells were harvested to a new centrifuge tube. Each tube had the same quantity of cells and the procedure followed manufacturer’s instructions. All staining procedures eliminated light contamination, and fluorescence intensity was quantified in a Tecan F200 microrespirometer. Results were presented by RFU. Each cell line was repeated three times.

### DNA sequencing and genetic analyses

Mitochondrial genome sequences of the three porcine cells (C1, C2 and C0) were analyzed by direct PCR and subsequent sequencing. PCR primers were listed in Table S1 as supplemental material. Sequences were assembled using DNASTAR Lasergene 10.1 software (http://www.dnastar.com/) and deposited at GenBank. Then SNPs among these three sequences were found out, and missense mutations were established the Grantham Score matrix referring to the amino acid difference formula^31^.

### Quantitation of mitochondrial genome copy number

Mitochondrial genome copy number was determined using ABI 7900 apparatus (ABI system, USA). The mtDNA specific primers (F: 5′-TATCCCTTATATCGGAACAG-3′, positions at 15797–15816; R: 5′-TGTTGGATCCGGTTTCGTGC-3′, positions at 15941–15960) were used to amplify the pig mtDNA were designed on the basis of the GenBank nucleotide sequence (NC_000845). The *GADPH* gene was used as the internal standard (F: 5′-GTCCACTGGTGTCTTCACGA-3′ and R: 5′-GCTGACGATCTTGAGGGAGT-3′). The product lengths were 164 bp (mtDNA fragment) and 154 bp (GADPH fragment) respectively. Purified products were cloned using pBLUE-T vector (Aidlab Co., China), and then were 10-fold serially diluted to prepare standard sample from 10^8^ to 10^3^ copy numbers.

### Cluster analyses

Mitochondrial DNA control region and genome sequences generated in this study and corresponding sequences downloaded from GenBank were aligned using MEGA6 software^32^, consensus NJ trees were constructed by 1000 bootstraps also using MEGA6 software.

## Additional Information

**How to cite this article**: Yu, G. *et al.* Mitochondrial Haplotypes Influence Metabolic Traits in Porcine Transmitochondrial Cybrids. *Sci. Rep.* **5**, 13118; doi: 10.1038/srep13118 (2015).

## Supplementary Material

Supplementary Information

## Figures and Tables

**Table 1:** Missense mutations of mitogenomes from C0, C1 and C2.

Gene	Description	Gene position	Nucleotide change				AA change			Grantham Score
			**C0**	**C1**	**C2**	**AA position**	**C0**	**C1**	**C2**	
ND1	NADH dehydrogenase subunit 1	737	T	T	C	246	Phe	Phe	Ser	155
		754	T	T	C	252	Ser	Ser	Pro	74
		842	G	A	G	281	Arg	Gln	Arg	43
ND2	NADH dehydrogenase subunit 2	298	C	C	A	100	Leu	Leu	Met	15
		632	C	C	T	211	Thr	Thr	Met	81
		715	A	A	G	239	Ile	Ile	Val	29
		1006	A	A	G	336	Ile	Ile	Val	29
ATP8	ATP synthase F0 subunit 8	119	C	C	T	40	Thr	Thr	Ile	89
		188	C	C	T	63	Ser	Ser	Leu	145
		197	T	T	C	66	Leu	Leu	Pro	98
ATP6	ATP synthase F0 subunit 6	355	C	T	T	119	His	Tyr	Tyr	83
		407	C	T	T	136	Pro	Leu	Leu	98
		554	G	G	A	185	Ser	Ser	Asn	46
COIII	cytochrome c oxidase subunit III	95	T	T	C	32	Ile	Ile	Thr	89
ND3	NADH dehydrogenase subunit 3	22	T	T	C	8	Phe	Phe	Leu	22
		85	A	A	G	29	Thr	Thr	Ala	58
		283	C	A	C	95	Leu	Ile	Leu	5
		287	C	C	T	96	Thr	Thr	Ile	89
		340	A	A	G	114	Thr	Thr	Ala	58
ND4L	NADH dehydrogenase subunit 4L	37	A	A	G	13	Thr	Thr	Ala	58
		142	G	G	A	48	Val	Val	Ile	29
ND4	NADH dehydrogenase subunit 4	1081	A	A	G	361	Met	Met	Val	21
		1238	T	T	C	413	Ile	Ile	Thr	89
ND5	NADH dehydrogenase subunit 5	100	A	A	G	34	Asn	Asn	Asp	23
		592	T	T	C	198	Phe	Phe	Leu	22
		1196	C	C	T	399	Ala	Ala	Val	64
		1300	C	C	A	434	Gln	Gln	Lys	53
		1799	T	T	C	600	Met	Met	Thr	81
ND6	NADH dehydrogenase subunit 6	512	C	G	G	6	Gly	Ala	Ala	60
CYTB	cytochrome b	677	C	T	T	226	Thr	Ile	Ile	89
		883	A	A	G	295	Met	Met	Val	21
		940	G	G	A	314	Ser	Ser	Gly	56
