# Supplementary material for: Mitochondrial Haplotypes Influence Metabolic Traits in Porcine Transmitochondrial Cybrids
Source: Sci Rep. 2015 Aug 19;5:13118. doi: 10.1038/srep13118 (PMC4541322; doi:10.1038/srep13118)
Supplement: Supplementary Information [file srep13118-s1.pdf]

Supplementary material for

Mitochondrial Haplotypes Influence Metabolic Traits in Porcine Transmitochondrial Cybrids

Guanghui Yu[1*], Hai Xiang[1*], Jianhui Tian[1*], Jingdong Yin[2], Carl A. Pinkert[3], Qiuyan Li[4] and Xingbo Zhao[1]

1 National Engineering Laboratory for Animal Breeding; Ministry of Agricultural Key Laboratory of Animal Genetics, Breeding and Reproduction; College of Animal Science and Technology, China Agricultural University, 100193, Beijing, China

2 State Key Laboratory of Animal Nutrition, College of Animal Science and Technology, China Agricultural University, 100193, Beijing, China

3 Department of Pathobiology, College of Veterinary Medicine, Auburn University, Auburn, AL 36849, and Department of Biological Sciences, College of Arts and Sciences, The University of Alabama, Tuscaloosa, AL 35487, USA

4 State Key Laboratory for Agribiotechnology; College of Biological Sciences, China Agricultural University, 100193, Beijing, China

Correspondence and requests for materials should be addressed to X. Z. (zhxb@cau.edu.cn)

*These authors contributed equally to this work.

Table S1 Primer pairs for porcine mitochondrial DNA sequences.

Primer Pair No.	Primer sequences 5'-3'	Position*	Expectant size
1	F: AGGAGACTAACTCCGCCAT<br>R: CGCGGATACTTGCATGTGT	16556-16574<br>1289-1307	1365
2	F: ACTAAGTCAATGCCTATTCTG<br>R: CAAATGTATGAAACCTCAG	16298-16318<br>530-548	864
3	F: CTACACAATAACCTCCCATA<br>R:TGGCACGAGATTTACCAACT	1107-1126<br>1471-1490	384
4	F: GCTCATAACGCCTTGCTC<br>R:ATTCTTTCATCTTTCCCTT	1377-1394<br>2397-2415	1039
5	F: CACCTAGAAGATCCCACA<br>R:ACAACCAGCTATCACCAG	2239-2256<br>2617-2634	396
6	F: CCGTAAGGGAAAGATGAAAG<br>R:TATGGTTATTTTGACTGGT	2393-2412<br>3475-3493	1101
7	F: CCGTGCAAAGGTAGCATA<br>R:CCAACATCGAGGTCGTAA	3189-3206<br>3589-3606	418
8	F: TGGGGTGACCTCGGAGTAC<br>R:AATATGGCGAAAGGTCCGG	3423-3441<br>4571-4589	1167
9	F: CGAGCAGTAGCCCAAACA<br>R:GGTCGTATCGGAATCGTG	4321-4338<br>4754-4771	451
10	F: GTATCAGGCTTTAACGTAGA<br>R:TGGTAATACTGCTGTCATTC	4543-4562<br>5652-5671	1129
11	F: CACAGAAGCAGCCACAAA<br>R:ATGGGATAGGGATAAAGT	5242-5259<br>5765-5782	541
12	F: ACATAGGATGAATGACAGC<br>R:TGGTGGAAGTAGTCAGAAAC	5643-5661<br>6812-6831	1189
13	F: GCACTGCCTTGAGCCTAC<br>R:GTGTTCAGGTTGCGGTCT	6599-6616<br>7143-7161	563
14	F: CTGACTCGTACCGCTAATA<br>R:ATGCAAATGCTTCTCAGAT	6750-6768<br>7924-7942	1193
15	F: CACTTTGTAATCATATTCGTAG<br>R:TAGTTGGAAAGGGTAAGC	7747-7768<br>8206-8223	477
16	F: TTCATCTCACTAACAGCAG<br>R:TTGAGTTCGGTTGATTCTG	7885-7903<br>9068-9086	1202
17	F: GCTTCATGCCCATTGTAC<br>R:TTATAGCGGAATCCTGTG	8816-8833<br>9461-9478	663
18	F: GCAAGCCCAGAATCAACCG<br>R:CGAGGAGGATTGAGGTGTT	9061-9079<br>10196-10214	1154
19	F: ATACCACATAGTAAACCCAA<br>R:CCTGTAGCCACAAAGAAA	9820-9839<br>10387-10404	585
20	F: CTAAACACCTCAATCCTCC<br>R:TTGGACGTAATCGGTACCG	10193-10211<br>11323-11341	1149
21	F: CCTTGCAGGGTTACTTAT<br>R:TTCGGGTTGTGGTTTCTT	11113-11130<br>11615-11632	520
22	F: CGGTACCGATTACGTCCAA<br>R:CCGATTAGATTGATGGATG	11323-11341<br>12470-12488	1166
23	F: ACCAGCTCTATCTGCTTA	12172-12189	473

	R:GAGGCTTTGATGTTGTTA	12627-12644	
24	F: ATGATGACTAATAGCAAGCC	12429-12448	1199
	R:GGGATGTAGTCCGAATTG	13610-13627	
25	F: CATCGGAGACATTGGATT	13462-13479	402
	R:AGTTGGCTTGAAGTTGAG	13846-13863	
26	F: CCTACTCCTAGCTGCAGCAG	13579-13598	1187
	R:ATTATGGAGATTACTCGTGG	14746-14765	
27	F: TCCGCATCATCATTACTA	14576-14593	612
	R:TTTATGGTGGACTTGGGT	15170-15187	
28	F: TAATTACCACGAGTAATCTC	14740-14759	1088
	R:TTCTACGAGGTCTGTTCCG	15809-15827	
29	F: GGAGCATCCATATTCTTT	15597-15614	516
	R:GGTGTAGTTGTCTGGGTCT	16094-16112	
30	F: TCGTAGAATGAATCTGAGG	15820-15838	1095
	R:GGTGATACGCATGTTGACTG	282-301	

*Positions are relative to the NC_000845 sequence.

Table S2 Genetic variations of mitogenome sequences among Lantang, Xiang and Large White swine breeds.

Gene	Reference position*	Lantang	Xiang		Large White		AA change
		KC250274 (C0)	EF545593	KC250273 (C1)	KC250275 (C2)	AP003428	
Control Region¶	109	C	C	C	T	T	
	124	C	C	C	T	T	
	131	A	A	A	G	G	
	136	-	-	-	C	C	
	145	T	T	T	C	C	
	153	T	T	T	C	C	
	158	G	G	G	A	A	
	181	C	C	C	T	C	
	241	C	C	T	T	T	
	277	A	G	A	A	A	
	279	C	T	C	T	C	
	294	G	G	G	A	A	
	301	C	C	T	C	C	
	306	T	T	T	C	C	
	323	T	T	T	C	T	
	390	T	T	T	C	C	
	405	C	T	T	T	T	
	452	T	C	T	C	C	
	462	C	C	C	C	G	
	560	T	T	C	T	T	
	575	G	G	G	A	A	
	704	G	G	G	A	A	
	706	G	G	G	A	A	
	870	C	C	C	T	C	
	1089	C	C	C	T	T	
	1096	G	G	G	A	A	
	1146	C	C	C	T	T	
	1167	T	T	T	C	T	
tRNA-Phe	1225	G	G	G	A	A	
12S	1236	C	C	C	T	T	
	1333	C	C	C	T	T	
	1559	C	C	C	T	T	
	1910	A	A	A	G	G	
	1984	C	C	C	T	T	
	1991	T	T	T	C	C	
tRNA-Val	2255	T	T	T	C	C	
16S	2259	A	A	A	G	G	
	2294	T	T	T	C	C	
	2534	T	T	T	C	C	
	2985	T	T	T	C	C	
	3009	G	G	G	A	A	
	3023	T	T	T	C	C	
	3287	G	G	G	A	A	
	3355	C	T	T	T	T	
	3372	T	T	T	C	C	
	3561	A	A	A	G	G	

	3794	A	A	A	T	T	
tRNA-Leu	3920	G	G	G	A	A	
ND1	3936	C	C	C	T	T	
	3951	C	C	C	T	T	
	4002	A	A	A	G	G	
	4218	T	T	T	T	C	
	4263	T	T	T	C	C	
	4290	T	T	T	C	C	
	4341	T	T	T	C	C	
	4359	G	G	G	A	A	
	4380	G	G	G	A	A	
	4392	T	T	T	C	T	
	4410	C	C	C	A	A	
	4632	C	C	C	T	T	
	4658	**T**	**T**	**T**	**C**	**C**	Phe/Ser
	4675	**T**	**T**	**T**	**C**	**C**	Ser/Pro
	4763	**G**	**G**	**A**	**G**	**G**	Arg/Gln
	4767	T	T	T	C	C	
	4860	T	T	T	C	C	
ND2	5089	A	A	A	T	T	
	5128	A	A	A	G	G	
	5290	G	G	G	A	A	
	5384	**C**	**C**	**C**	**A**	**A**	Leu/Met
	5473	A	A	A	G	G	
	5549	T	T	T	C	C	
	5557	G	G	G	A	A	
	5593	A	A	A	G	G	
	5599	C	C	C	T	T	
	5629	T	T	T	C	C	
	5674	T	T	T	C	C	
	5718	**C**	**C**	**C**	**T**	**T**	Thr/Met
	5794	G	G	G	A	A	
	5810	**A**	**A**	**A**	**G**	**G**	Ile/Val
	5869	A	A	A	G	G	
	5884	C	C	C	T	T	
	6013	T	T	T	C	C	
	6059	C	C	C	T	T	
	6085	G	G	G	A	A	
	6092	**A**	**A**	**A**	**G**	**G**	Ile/Val
tRNA-Ala	6217	T	T	T	C	C	
	6219	C	C	C	T	T	
tRNA-Cys	6429	C	C	C	T	T	
COI	6846	A	A	A	T	T	
	6873	G	G	G	A	A	
	6891	C	C	C	T	T	
	6930	A	A	A	G	G	
	6943	C	C	C	T	T	
	7029	T	T	T	C	C	
	7242	T	T	T	C	C	

	7260	G	G	G	A	A	
	7368	C	C	C	T	T	
	7407	G	G	G	A	A	
	7434	C	C	C	T	T	
	7590	C	C	C	T	T	
	7671	T	T	T	C	C	
	7758	T	T	T	C	C	
	7938	T	T	T	C	C	
gap	8056	G	G	A	G	G	
tRNA-Asp	8188	A	G	G	G	G	
COII	8292	T	T	T	C	C	
	8334	T	T	T	C	C	
	8419	T	T	T	C	C	
	8526	T	T	T	C	C	
	8634	G	G	G	A	A	
	8664	G	G	G	A	A	
	8682	A	A	A	G	G	
	8694	T	T	T	C	C	
ATP8	8979	T	T	T	C	C	
	9077	C	C	C	T	T	Thr/Ile
	9078	C	C	C	T	T	
	9146	C	C	C	T	T	Ser/Leu
	9155	T	T	T	C	C	Leu/Pro
ATP6	9146	C	C	C	T	T	
	9155	T	T	T	C	C	
	9293	G	A	A	A	A	
	9356	T	T	T	C	C	
	9474	C	C	T	T	T	His/Tyr
	9526	C	C	T	T	T	Pro/Leu
	9673	G	G	G	A	A	Ser/Asn
	9710	C	C	C	T	T	
COIII	9838	A	A	T	A	A	
	9894	T	T	T	C	C	Ile/Thr
	9991	A	A	A	G	G	
	10021	C	C	C	T	T	
	10405	A	A	A	G	G	
	10450	G	G	G	A	A	
tRNA-Gly	10601	C	C	C	T	T	
ND3	10674	T	T	T	C	C	Phe/Leu
	10737	A	A	A	G	G	Thr/Ala
	10865	C	C	C	T	T	
	10935	C	C	A	C	C	Leu/Ile
	10939	C	C	C	T	T	Thr/Ile
	10992	A	A	A	G	G	Thr/Ala
ND4L	11083	C	C	C	T	T	
	11105	A	A	A	G	G	Thr/Ala
	11110	T	T	T	C	C	
	11180	T	T	T	C	C	

	11210	**G**	**G**	**G**	**A**	**A**	Val/Ile
	11248	C	C	C	T	C.	
	11287	G	G	G	A	A	
	11293	A	A	A	G	G	
	11353	C	C	C	T	T	
ND4	11604	C	C	C	T	T	
	11707	T	T	T	C	C	
	11751	C	C	C	T	T	
	11865	C	C	C	T	T	
	11985	A	A	A	G	G	
	12030	T	T	T	C	C	
	12162	T	T	T	C	C	
	12219	T	T	T	C	C	
	12291	A	A	A	G	G	
	12390	A	A	A	G	G	
	12439	**A**	**A**	**A**	**G**	**G**	Met/Val
	12504	T	T	T	C	C	
	12570	T	T	T	C	C	
	12596	**T**	**T**	**T**	**C**	**C**	Ile/Thr
tRNA-Leu	12879	G	G	G	A	A	
ND5	12883	T	T	T	C	C	
	12970	G	G	G	A	A	
	13034	**A**	**A**	**A**	**G**	**A**	Asn/Asp
	13174	T	T	T	T	C	
	13354	C	C	C	A	A	
	13393	C	G	G	G	G	
	13399	C	C	C	T	T	
	13502	T	T	T	C	C	
	13526	**T**	**T**	**T**	**C**	**C**	Phe/Leu
	13759	C	C	C	T	T	
	13915	T	T	T	T	C	
	13918	T	T	T	C	C	
	14119	C	T	C	C	C	
	14130	**C**	**C**	**C**	**T**	**T**	Ala/Val
	14134	C	C	C	T	T	
	14234	**C**	**C**	**C**	**A**	**A**	Gln/Lys
	14320	C	C	C	T	T	
	14482	A	A	A	C	C	
	14560	G	G	G	A	A	
	14659	T	T	T	C	C	
	14733	**T**	**T**	**T**	**C**	**C**	Met/Thr
ND6	14760	C	C	C	T	T	
	14868	T	T	T	C	C	
	14919	A	A	A	G	G	
	14946	T	T	T	C	C	
	15030	G	G	G	A	A	
	15048	G	G	G	A	A	
	15072	T	T	T	C	C	
	15141	A	G	A	A	A	

	15198	T	T	T	C	C	
	15250	C	G	G	G	G	Gly/Ala
	15258	T	T	T	C	C	
tRNA-Glu	15283	C	C	C	T	T	
	15548	C	C	C	T	T	
	15584	C	C	C	T	T	
	15608	G	G	G	A	A	
	15644	C	C	C	T	T	
	15884	T	T	T	C	C	
	15933	C	C	C	C	A	Leu/Met
	16018	C	T	T	T	T	Thr/Ile
	16034	G	G	G	A	A	
	16181	C	C	C	T	T	
CYTB	16215	C	C	C	T	T	
	16217	A	A	A	G	G	
	16220	T	T	T	C	C	
	16224	A	A	A	G	G	Met/Val
	16253	G	G	G	A	G	
	16262	A	A	A	A	G	
	16281	A	A	A	G	G	Ser/Gly
	16379	A	A	A	G	G	
	16415	T	T	T	C	C	
	16475	G	G	G	A	A	
tRNA-Thr	16487	T	T	T	C	C	
	16531	A	A	A	G	G	

Note: * NC_000845 sequence reference positions. ¶Compared to the reference sequence, DNA sequence AP003428 of Large White swine exceed 16 motifs of 10-bp tandem repeat sequence (TACACGTGCG), and sequence KC250273 and EF545593 of Xiang swine exceed 10 motifs and 8 motifs of the same 10-bp tandem repeat sequence, respectively. In addition, Lantang sequence KC250274 contains an 11-bp insertion (TAAAACACTTA) between the referenced positions 1013 and 1014.
